# Potential Landscape and Probabilistic Flux of a Predator Prey Network

**DOI:** 10.1371/journal.pone.0017888

**Published:** 2011-03-15

**Authors:** Chunhe Li, Erkang Wang, Jin Wang

**Affiliations:** 1 State Key Laboratory of Electroanalytical Chemistry, Changchun Institute of Applied Chemistry, Chinese Academy of Sciences, Changchun, Jilin, China; 2 Department of Chemistry and Physics, State University of New York at Stony Brook, Stony Brook, New York, United States of America; 3 Graduate School of the Chinese Academy of Sciences, Beijing, China; University of Calgary, Canada

## Abstract

Predator-prey system, as an essential element of ecological dynamics, has been recently studied experimentally with synthetic biology. We developed a global probabilistic landscape and flux framework to explore a synthetic predator-prey network constructed with two Escherichia coli populations. We developed a self consistent mean field method to solve multidimensional problem and uncovered the potential landscape with Mexican hat ring valley shape for predator-prey oscillations. The landscape attracts the system down to the closed oscillation ring. The probability flux drives the coherent oscillations on the ring. Both the landscape and flux are essential for the stable and coherent oscillations. The landscape topography characterized by the barrier height from the top of Mexican hat to the closed ring valley provides a quantitative measure of global stability of system. The entropy production rate for the energy dissipation is less for smaller environmental fluctuations or perturbations. The global sensitivity analysis based on the landscape topography gives specific predictions for the effects of parameters on the stability and function of the system. This may provide some clues for the global stability, robustness, function and synthetic network design.

## Introduction

Ecological systems exist in a self sustainable way within which the elements are interacting with each other and with outside environments there are constant energy, information and material exchanges. To perform biological functions, the ecosystems need to be stable. Therefore global stability is essential for ecosystems. The challenge is how to quantify the global stability. There have been increasing numbers of studies on the global topological structures of the network systems, recently [1]. The underlying nature of networks has been explored by many experimental research [2]. However, there are very few studies about why the networks are robust and perform their biological functions from the physical point of view.

In the cell, statistical fluctuations from a finite number of molecules provide the source of intrinsic noise, and highly dynamical and inhomogeneous environments provide the source of external noise for the networks. So, we should study the network dynamics in fluctuating conditions in order to model realistically the cellular inner and outer environments. The dynamics with extrinsic fluctuations can be described by probability diffusion equations. For dynamics with intrinsic fluctuations, master equations [3] can provide the description.

The conventional methods of describing the networks according to deterministic or stochastic chemical kinetics often explore only the local properties of the networks [4], [5]. Here, we will explore the global nature of the network from physical perspectives, formulating the problem in terms of probabilistic landscape and flux framework. Networks have huge state space. Why seemingly infinite number of state space (for example, genotypes in gene regulation networks) can result in a finite of number of functional states (for example, phenotypes from gene regulations)? Probabilistic description may provide an answer because every state has different weight. Functional states may correspond to higher probability ones and occupy lower potential valleys [6]–[16]. Furthermore, the dynamics of the network can be decomposed of the gradient of the landscape and the curl flux flow [14]–[16]. Using this framework, the global stability and robustness of the networks can be explored and further quantified in terms of the topography of the underlying probabilistic landscape.

In this paper we employed a predator-prey network which is constructed using two Escherichia coli populations [17]. As an essential component of ecological dynamics, natural predator-prey systems have been studied extensively by experiments and modeling [18], [19]. Compared with other types of ecological interactions such as mutualism and competition, predation often generates richer dynamics and so gives a greater challenge to engineer de novo [17]. Recently there has been experimental studies on interacting Escherichia coli populations, synthetic ecosystems — using genetic regulatory networks and intercellular communications systems to control and coordinate the behavior [17], [20], [21]. The two E. coli populations of this system, communicate and control each others population density by producing small-molecule signals (AHLs) that can diffuse across cell membranes into the medium and regulate gene expression. The basic logic is similar to a predator-prey system: without the ‘prey’, the ‘predator’ population decays at a high rate due to expression of a lysis gene it carries. As the prey grows, it produces an AHL that diffuses through the medium into the predator, where it rescues the predator by inhibiting lysis gene expression. The predator produces a second AHL that diffuses into the prey and initiates synthesis of the lysis gene, effecting ‘predation’. The mathematical model for the system can be reduced to four differential equations of the average populations for the predator and prey as well as the effects of the concentration and lethality of the lysis protein in the corresponding cell.

We explore the corresponding probabilistic diffusion equation and uncover the underlying landscape and flux with self consistent mean field method. The theoretical studies can provide detailed guidance for experimental implementation. They will highlight the importance of controlling the expression, lethality, and stability of the lysis proteins. The function of a genetic circuit could be optimized by directed evolution [17], [20], [21], and will allow us to efficiently explore circuit function in different regions of the parameter space. Synchronization of intra-cellular behavior across a population, achieved by inter-cellular communication [17], [20], [21], may render the circuit more resistant to fluctuation in individual cells.

By varying biologically feasible parameter values, we will quantitatively predict whether and when the circuit will generate stable oscillations in population densities and intracellular gene expressions in fluctuating environments, which will be directly tested from the experiments. In addition, the theoretical prediction and experimental (test) validation will uncover the key design features and topological structure of the underlying landscape required to achieve the target circuit function in an experimental system. Through the analysis on the underlying landscape, we can also understand more clearly the sensitivity of the parameters on the stability of the system.

## Results and Discussion

### Probabilistic Landscape and Flux

**Figure 1:**
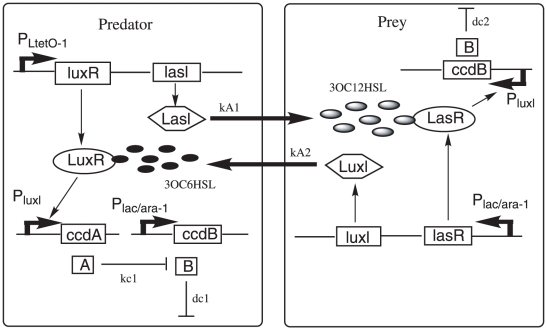
A synthetic predator-prey ecosystem diagram. Outer boxes represent cell walls. Arrows represent activation or production, blunt arrows represent inhibition or killing.

Figure 1 shows an illustration of the predator-prey synthetic ecosystem. In this system, predator and prey communicate and regulate each other's density. When prey density is low, a suicide gene(ccdB) is continuously expressed, making predator density repressed. When prey density increases, an acyl-homoserine lactone(AHL), 3OC6HSL, is activated in prey cell. When it reaches sufficiently high concentrations, it is bound to the transcriptional regulator *LuxR* in the predator cells, which leads to the expression of an antidote gene(ccdA) and then rescue of predator cells. In addition, when predators increase, they produce another AHL, 3OC12HSL, which enters into the prey cells and activates expression of ccdB gene, causing ‘predation’ [17].

**Figure 2:**
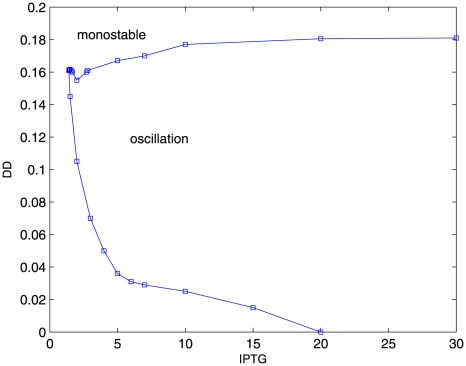
The phase plane portrait for the predator-prey network in terms of parameter *IPTG* and *DD*.

For illustration purpose, we fix all other system parameters except *DD* (dilution rate) and *IPTG* (isopropyl-*β*-thiogalactopyranoside) which promote predator and prey interactions. Figure 2 shows the phase plane of system in terms of parameter *IPTG* and *DD* from the analysis of the deterministic equations. We can see that the system has two phase regions: an unstable limit cycle oscillation phase and a mono-stable phase. When a set of parameters are specified as: *IPTG* = 5, *DD* = 0.1125, the fixed point is unstable and a limit cycle emerges.

Employing the self consistent mean field approximation, after obtaining the solutions of the mean and variance for 4 variables, we can acquire the probability distribution 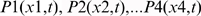 for every single variable by gaussian approximation discussed in Methods part. Then we can solve the steady state probability distribution *P* for the density of the predator-prey network given diffusion coefficient *D*. From the steady state distribution results, we can identify 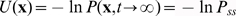 ( when 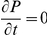) [8]–[15]. In this way, we can map out the potential energy landscape *U*. For predator-prey network with 4 variables, in order to visualize the results conveniently, we select two variables to illustrate the results by integrating out the other 2 variables. Here we choose two variables x3(3OC12HSL) and x4(3OC6HSL) to compute their probability distribution. And the corresponded probability distribution is: 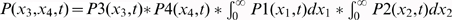.

For nonequilibrium system, the driving force *F* can not be written as the gradient of potential *U*, like the equilibrium case. In general, *F* can be decomposed into a gradient of a potential and a curl flow flux [14], [15] 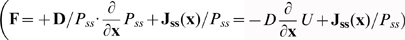. *P_ss_* represent steady state probability distribution and potential U is defined as 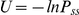. And the probability flux vector **J** of the system in concentration space **x** is defined as [3]: 
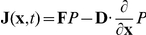
.

The diffusion equation with constant diffusion coefficient *D* can be written as 
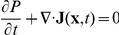
. In steady state, 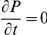, then 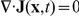. The divergent free flux implies the rotational nature of the steady state flux field **J**_*ss*_. Here, something needs to be noted. The divergence of **J**_*ss*_ is zero, however the divergence of **J_ss_**/*P_ss_* is in general not zero with finite noise strength. Only in the situation when the **J**_*ss*_ is perpendicular to the landscape gradient 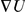 (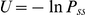), the divergence of **J_ss_**/*P_ss_* is equal to zero. This happens when the noise strength is approaching zero. So in the low noise, our decomposition is equivalent to Helmhotz decomposition.

**Figure 3:**
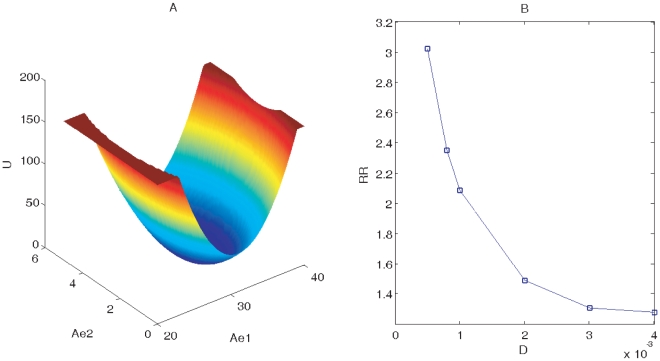
Landscape for monostable state. (A) shows 3-dimensional Landscape for monostable state at D = 0.001, IPTG = 5, DD = 0.02 using variables *Ae*1 and *Ae*2. (B) shows RR (robustness ratio) versus external noise D for monostable state using the same parameters values.

When parameters are specified as: *IPTG* = 5, *DD* = 0.02, from the phase plane we can see that system is in the monostable state. Figure 3(A) shows 3 dimensional landscape for monostable state using the last parameters at small fluctuations *D* = 0.001. From the figure, we can see that there is one stable local minimum or attraction of basin, corresponding to the coexistence state of predator and prey. This shows that system is attracted to one stable point and the monostable state is stable in small external fluctuations.

**Figure 4:**
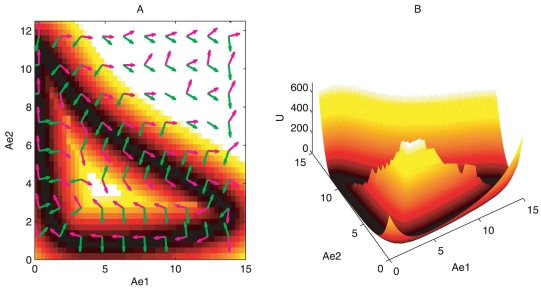
Landscape and probabilistic flux for oscillation state. (A) shows the 2-dimensional landscape and probabilistic flux for oscillation state at D = 0.001, IPTG = 5, DD = 0.1125. Magenta arrows represent the flux flow vector, green arrows represent the negative gradient of potential energy. (B) shows the 3-dimensional landscape for predator prey network.

Figure 4 shows 2 and 3 dimensional landscape for oscillation state at *D* = 0.001 when parameters are given by *IPTG* = 5, *DD* = 0.1125. From Figure 4 we can see that the closed ring is around the deterministic oscillation trajectory. This means the potential is lower (corresponded to higher probability) along the oscillation path or on the closed ring. Inside the closed ring, the potential is higher forming a mountain or hat. Outside the closed ring, the potential is also higher. The system is therefore attracted to the closed ring rather than a particular stable basin. Furthermore, the probability flux is plotted. We can see that outside the ring valley, the dynamics is determined by mostly the gradient of the potential landscape. But on the ring, the dynamics is mostly controlled by the curl probability flux to maintain the coherence of the oscillations [14], [15]. Both probability landscape and flux vector are paramount in determining the stable oscillation. The potential landscape attracts the system down to the closed oscillation ring, while the probability flux drives the system move periodically along the oscillation ring.

### Robustness Ratio, Global Stability, Barrier Height, and Entropy Production Rate

Having the underlying potential landscape, we can further study the global stability and robustness of system at different fluctuation strengths characterized by the diffusion coefficient *D* through computing the barrier height for oscillation and robustness ratio *RR* for monostability.

For monostability, we define robustness ratio *RR* for the network as 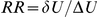 to quantify global stability. Here the *δU* is the difference between the global minimum of *U* and the average of *U*, 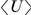, and Δ*U* is the variance or half width of the distribution of *U*. The *δU* characterizes the bias or the slope toward the global minimum of the potential landscape, while Δ*U* is a measure of the averaged roughness or the local trapping of the potential landscape. Figure 3(B) shows that the global stability measured by *RR* decreases when external noise increases. This means under fluctuations the monostable system will become less stable. Less fluctuations lead more robust networks.

**Figure 5:**
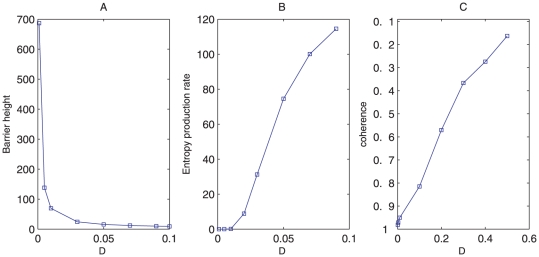
Barrier height, entropy production rate and phase coherence at different diffusion coefficient *D*. (A), (B), (C) show barrier height, entropy production rate and coherence versus diffusion coefficient *D* separately.

For oscillation state, we define the barrier heights *Umax-Umin* as the global stability measure. *Umin* is the potential minimum along the limit cycle attractor. *Umax* is the potential at the local maximum point inside the limit cycle circle (the top of the Mexican hat). In Figure 5A, as the diffusion coefficient characterizing the fluctuations decreases, the barrier heights related with escaping from the limit cycle attractor increases. The resulting limit cycle attractor becomes more stable. Therefore, small fluctuations and large barrier heights lead to robustness and stability in the oscillatory network [14], [15].

Additionally, we compute entropy production rate or dissipation cost for different fluctuations [22]. Figure 5B shows that entropy production rate increases as diffusion coefficient *D* characterizing the fluctuations increases. This implies that nature might evolve such that the network is robust against environmental perturbations, and performs specific biological functions with minimum dissipation cost. In our study, this is also the equivalent of optimizing the global stability and robustness of the network [16].

### Amplitude, Period and Coherence

**Figure 6:**
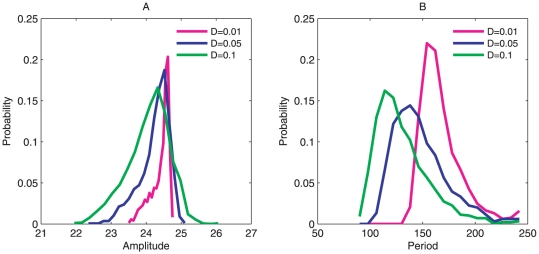
Distribution of amplitude and period. (A), (B) show the distribution of amplitude and period at different diffusion coefficient *D* separately.

In addition, we also used method of the stochastic dynamics to learn more of the global stability and robustness of the oscillations under different fluctuations. We followed the stochastic Brownian dynamics rather than the deterministic average dynamics. Figure 6 shows the distributions of the period and amplitude of oscillations for variable x3(3OC12HSL) at different diffusion coefficient *D*. We can see that the distribution for amplitude and period become more spread out when the fluctuations increase. The standard deviation σ from the mean increases and more other possible values of the amplitude and period of oscillations can appear when the fluctuations increase [23]. This implies that less fluctuations produce more stable network and make more coherent oscillations with less number of possible value of amplitudes and period.

We also obtained the coherence *ξ*, which measures the degree of periodicity of the time evolution of a given variable [24], at different diffusion coefficient *D*. In the presence of fluctuations, the more periodic the evolution is, and the larger value of *ξ* appears. In Figure 5C, *ξ* decreases when the diffusion coefficient increases. This means larger fluctuations tend to destroy the coherence of the oscillations and also the robustness of the system.

### Sensitivity Analysis

**Figure 7:**
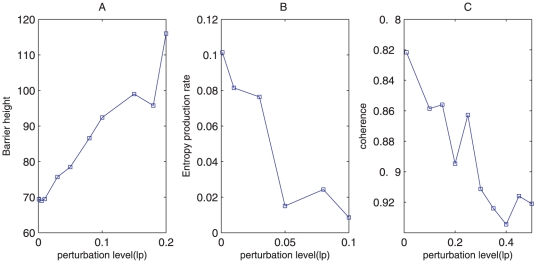
Barrier height, entropy production rate and phase coherence at different perturbation of parameters. (A), (B), (C) show barrier height, entropy production rate and coherence versus perturbation level(*lp*) separately.

For oscillation state we also explore the effects of parameters on the stability and robustness of system by measuring the changes of barrier heights after giving parameters a perturbation level *lp*. From Figure 7, we can see that barrier height increase, the entropy production rate decrease, and coherence increase as the perturbation level(*lp*) of the chemical reaction rates constants increase. This shows that the current parameters are not the ones which make system most stable.

**Figure 8:**
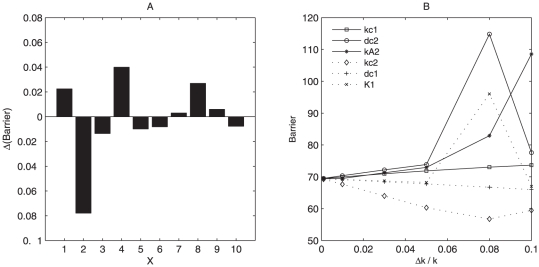
Sensitivity analysis. (A) shows the effects of parameters on the barrier height at the same perturbation. x axis represent: 1:kc1, 2:kc2, 3:dc1, 4:dc2, 5:K1, 6:K2, 7:kA1, 8:kA2, 9:dAe1, 10:dAe2. (B) shows respectively the effect of 6 parameters on barrier height. Δ*k/k* represents the percent of parameters increased.

Therefore, we further did the sensitivity analysis of different parameters by giving a percentage Δ*k/k* as the degree for change. Figure 8(A) shows the effects of some parameters on the barrier heights measuring stability of system. We selected some top important parameters and then studied the effects of them on robustness of the oscillation system as shown in Figure 8(B). We can see that the parameter *kA*2,*kc*1,*dc*2 give the positive contribution to the stability of the system. It means when these parameters increase, the system becomes more stable. However, the parameter *dc*1,*kc*2 give the negative effects on the stability of system. It means when these parameters increase, the system becomes less stable. Here *kc*1,*kc*2 are growth rates of predator and prey, and *dc*1, *dc*2 are cell death rates of predator and prey separately. *kA*1 is the synthesis rate of AHL(acyl-homoserine lactone) by predator(3OC12HSL), *kA*2 is the synthesis rate of AHL by prey(3OC6HSL). *kA*2 gives the positive contribution to the stability of system could be well explained because 3OC6HSL is to rescue predator by initiating *ccdA* expression, and its activity promotes the mutual regulation of predator and prey. Therefore this makes the predator prey oscillation dynamics more stable, which is consistent with the experimental conclusions about the effects of AHL on the system [17].

For *dc*2, from the Figure 1, we can see *dc*2 characterize the ability of predation, so, *dc*2′ effects on the stability of system could be explained. For the oscillation system based on current parameters, prey is a little dominant, increasing *dc*2 means increasing the repression of predator to prey. In this way, the system is more inclined to equilibrium for two species, and so becomes more stable. However, when *dc*2 increases to some extent, predator and prey have been in equilibrium, and at this time increasing it further more will destroy the oscillation state. Figure 8(B) shows that the stability of the system increases first and then decreases as *dc*2 increases, which is consistent with the above analysis.

For the parameter *kc*1 and *dc*1, they have opposite effects on predator density. *kc*1 strengthens predator, and *dc*1 weakens predator. In the similar way, the effects of these two parameters on the stability of system could be explained. Since in the system with the current parameters prey are more dominant, increasing *kc*1 will activate predator by repressing *ccdB*, and promote the equilibrium of density for two species, which is reflected by the more stable oscillation dynamics. Therefore, the increase of *kc*1 increases the stability of the system quantified by the barrier heights of oscillation system. For *dc*1 and *kc*2, these two parameters promote the prey and inactivate predator, so activation of *dc*1 and *kc*2 decrease the stability of system. The sensitivity analysis results give specific predictions on the parameter changes on global stability and can provide some clues for the experimental validation and test. It will also give some insights for the de novo design of synthetic predator-prey network.

### Conclusions

We explored the global nature of a predator-prey network in terms of the potential landscape with a self consistent mean field approximation method. We used the experimentally inferred rate parameters to explore the system by computing the landscape topography characterized by barrier heights. This provides a quantitative measure for the stability of oscillation system. The entropy production rate results imply that nature might evolve such that the network is robust against internal and environmental perturbations, and performs specific biological functions with minimum dissipation cost.

The landscape of the oscillation network has a closed ring valley shape attracting the system down. The landscape and the probabilistic flux determine the dynamics of the nonequilibrium oscillation system together. The landscape drives the system toward the ring valley, and the flux vector makes the system move along the oscillation ring. Therefore, Mexican hat like landscape topography provides an optimal criterion to select the suitable parameter subspace of network, guarantee the stability and robustness with less dissipation cost and perform specific biological functions, which is useful for the network design. Our approach is general and can be applied to other complicated protein networks and gene regulatory networks, to explore the underlying global potential landscape.

By the sensitivity analysis of biologically feasible parameter, we quantitatively predict the effects of parameters on the stability of the oscillation system in population densities and intracellular gene expressions in fluctuating environments, which will be directly tested from the experiments. Additionally, the theoretical prediction and experimental validation will uncover the key design features and topological structure of the underlying landscape required to achieve the target circuit function in an experimental system. The synthetic ecosystems will serve as well-defined systems for exploring evolutionary and ecological questions like the generation and maintenance of biodiversity and the role of programmed cell death in bacteria [17], [20], [21]. This will allow us to explore the interplay between environment, gene regulation and population dynamics, the central issue of ecology.

## Methods

In order to uncover the probability landscape, we begin from the chemical reaction network involved in predator-prey network. The statistical nature of the chemical reactions can be captured by the corresponding diffusion equation, which describe the evolution of the networks probabilistically. The diffusion equation is hard to solve due to its inherent huge dimensions. We therefore used the self consistent mean field approximation to reduce the dimensionality [8], [13]. In this way, we could follow the time evolution and steady state probability of the protein concentrations. From the steady state probability we can get the potential energy landscape.

### The Predator Prey Network of 4 Variables

The ordinary differential equations for the predator prey system can be written as follows [17]:
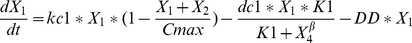


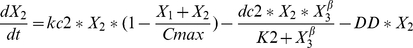


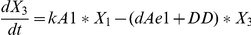


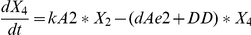



Here *X*_1_, *X*_2_ represent separately density of predator and prey, *X*_3_ is the concentration of 3OC12HSL, *X*_4_ is the concentration of 3OC6HSL. The first two equations describe the cell populations, and the last two equations describe the levels of the AHLs in the medium. And the the meaning and range of parameters are described in Table 1.

**Table 1:** Parameter values of predator prey model.

Parameter	Description	Base value
*kc*_1_	Predator cell(MG1655) growth rate constant	0.8 hr
*kc*_2_	Prey cell(Top10F′) growth rate constant	0.4 hr
*Cmax*	Carrying capacity for cell growth	100×10^3^ *cells nL* ^−1^
*β*	Cooperativity of AHL effect	2
*dc*_2_	Prey cell death rate constant	0.3 hr^−1^
*dc*_1_	Predator cell death rate constant	0.5+1×*IPTG* ^2^/(5^2^+*IPTG* ^2^)
*K*1,*K*2	Concentration of AHL necessary to half-maximally active *P_luxI_* promoter	10 nM
*kA*_1_	Synthesis rate constant of AHL by the predator cell	0.1 nM ml hr^−1^
*kA*_2_	Synthesis rate constant of AHL by the prey cell	0.02+0.03×*IPTG* ^2^/(5^2^+*IPTG* ^2^)
*dAe*_1_	Decay rate constant of 3OC12HSL in the cell	0.017 hr
*dAe*_2_	Decay rate constant of 3OC6HSL in the cell	0.11 hr
*DD*	Dilution rate	0–0.3 hr^−1^

*DD* is a dilution rate and calculated with the relation 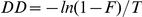 where F is a fraction of dilution and *T* is the time between each dilution event [27].

### Self Consistent Mean Field Approximation

The diffusion equations are the equations for the time evolution of the probability of some specific state 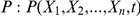, where 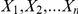 is the concentration or populations of molecules or species. We expected to have N-coupled differential equations, which are not feasible to solve. Following a self consistent mean field approach [8], [13], [16], we split the probability into the products of individual ones: 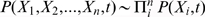 and solve the probability self-consistently. This effectively reduces the dimensionality from 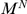 to 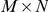, and therefore the problem is computationally tractable.

Although self consistent approximation reduces the dimensionality of the system, for the multi-dimension conditions, it is still hard to solve diffusion equations directly. We first consider moment equations. We can start from moment equations and then simply assume specific probability distribution based on physical argument, which means we give some specific relations between moments [16], [25]. In principle, once we know all moments, then we can construct the probability distribution. For example, Poisson distribution has only one parameter, so we may calculate all other moments from the first moment, mean. Here we use gaussian distribution as approximation, and then we need two moments, mean and variance.

When diffusion coefficient *D* is small, the moment equations can be approximated to [3], [6]:
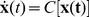
(1)

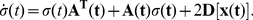
(2)


Here, **x**, σ(t), and **A**(*t*) are vectors and tensors, and **A^T^**(*t*) is the transpose of **A**(*t*). The matrix elements of A is 
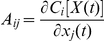
. According to this equations, we can solve **x**(*t*) and σ(t). We consider here only diagonal element of σ(t) from mean field splitting approximation. Therefore, the evolution of distribution for one variable could be obtained using the mean and variance by gaussian approximation:
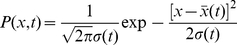
(3)


We can expand the results to the multi-dimensional system using the same method.

The probability obtained above corresponds to one fixed point or basin of attraction. One solution of the equations determines one of the fixed points and also gives the variation around the basin of attraction, so it is intrinsic. If the system allows multistability, then there are several probability distributions localized at every basin of attraction, but with different variations. Therefore, the total probability is the weighted sum of all these probability distributions. The weighting factors 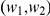 are the size of the basin, which represent the relative size of different basin of attraction. For example, for bistability 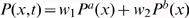, here 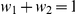.

While for oscillation, it is different from multistable states for obtaining the probability distribution. The mean and variance **x**(*t*) and σ(t), for oscillation are not constants even in steady state, they are functions of time. Here we obtained results by integration of the probability in time for one period and divide by the period : 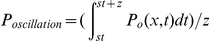.

Here, *z* is period of oscillation, and *st* is starting point for integration.

Finally, once we have the total probability, we can construct the potential landscape by the relationship with the steady state probability: 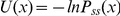. In the network system, every chemical parameter, such as synthesis and decay rates, will contribute to the structure and dynamics of the system. All these effects are encoded in the total probability distribution, and, consequently, the underlying potential landscape [13].

In the 4-dimensional protein concentration space, it's hard to visualize 4-dimensional probabilistic flux. However, the associated 2-dimensional flux vector for variable *x*_3_ and *x*_4_ can be acquired: 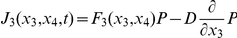 and 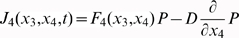.

Here, to compute 2-dimensional flux *J*_3_, *J*_4_ from 4-dimensional space, we adopted some approximation method in computation of the force *F*_3_ and *F*_4_, because generally *F* is the function of 4 variables(*x*_1_, *x*_2_, *x*_3_, *x*_4_). We project the 4-dimensional force *F* to 2-dimensional space(*x*_3_, *x*_4_). In this way, the force *F* can be transformed to the function of only two variables *x*_3_ and *x*_4_.

Therefore, like the computation of probability distribution *P*, the probabilistic flux vector also can be acquired by integration in one period:
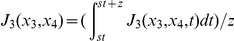


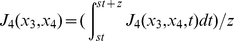
(4)


### Entropy Production Rate

For an non-equilibrium open system, there are constant exchanges in energy and information which result dissipations. The energy dissipation is a global physical characterization of the non-equilibrium system, and is closely related to the entropy production rate in the steady state. The entropy formula for the system is [22],
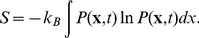
(5)


By differentiating the above equation, the increase of the entropy at constant temperature *T* can be acquired as follows:
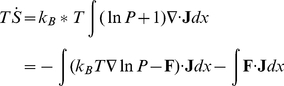
(6)where 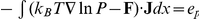 is the entropy production rate [22], and 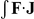 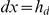 is the mean rate of the heat dissipation. In steady state 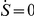, and the entropy production *e_p_* is equal to the heat dissipation *h_d_*. In this paper, we computed the heat dissipation rate and entropy production rate at steady state respectively and also validfied that they are the same numerically.

### Phase Coherence

**Figure 9:**
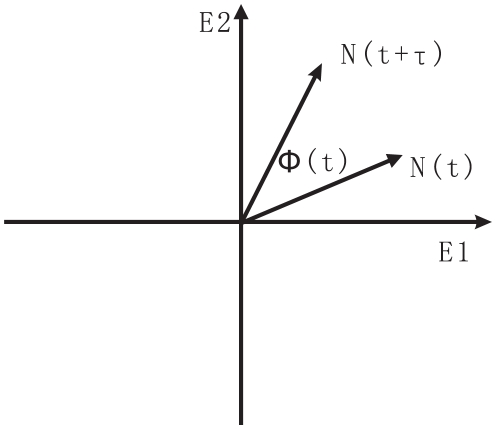
The definition of phase coherence.

The robustness and stability of the oscillation at different diffusion coefficient *D* can also be quantified by the phase coherence *ξ*, a measure of the degree of periodicity of the time evolution for a given variable [26]. The phase coherence *ξ* is defined as follows: First, the vector 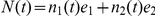 is shown in Figure 9. The unit vectors are 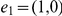 and 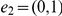, 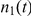 and 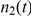 are the concentration of the two kinds of protein molecules or two species at time *t*. Then 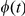 is the phase angle between *N*(*t*) and *N*(*t*+τ ), where τ should be smaller than the deterministic period and larger than the fast fluctuations. Here we choose τ = 2 *h*. 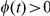 represents that the oscillation goes on the positive orientation (counterclockwise). The formula of *ξ* is: 
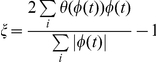
, where 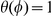 when 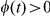, and 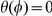 when 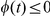, and sums are taken over every time steps for the simulation trajectories. 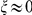 implies the system moves stochastically and has no coherence. The oscillation is most coherent when *ξ* is close to 1. In the presence of fluctuations, the more is *ξ*, the more periodic the evolution is.
